# Implementation of the WHO core components of an infection prevention and control programme in two sub-saharan African acute health-care facilities: a mixed methods study

**DOI:** 10.1186/s13756-023-01358-1

**Published:** 2024-01-15

**Authors:** R. Wood, W. Tembele, A. Hema, A. Somé, E. Kinganda-Lusamaki, C. Basilubo, R. Lumembe, N. Alama, G. Mbunsu, A. Zongo, S. Ahuka, J. J. Muyembe, F. Leendertz, T. Eckmanns, G. Schubert, T. Kagoné, S. Makiala, S. Tomczyk

**Affiliations:** 1 Robert Koch Institute, Berlin, Germany; 2 School of Public Health in Kinshasa, Hospital Saint Luc of Kisantu, Kisantu, Democratic Republic of Congo; 3 Centre Hospital University Sourou Sanou (CHUSS), Bobo-Dioulasso, Burkina Faso; 4 Centre Muraz, Bobo-Dioulasso, Burkina Faso; 5 Institut National de Recherche Biomédicale, Kinshasa, Democratic Republic of Congo; 6 Cliniques Universitaires de Kinshasa, Université de Kinshasa, Kinshasa, Democratic Republic of Congo; 7 Institute of Developmental Research, University of Montpellier, Montpellier, France; 8 Public health and Infection prevention control independent expert consultant, Kinshasa, Democratic Republic of Congo; 9 Helmholtz Institute for One Health, Greifswald, Germany

**Keywords:** Infection prevention and control programme, WHO core components, Healthcare-associated infections, Knowledge, Attitudes and practices (KAP), Training, Africa

## Abstract

**Background:**

The coronavirus pandemic again highlighted the need for robust health care facility infection prevention and control (IPC) programmes. WHO guidelines on the core components (CCs) of IPC programmes provides guidance for facilities, but their implementation can be difficult to achieve in resource-limited settings. We aimed to gather evidence on an initial WHO IPC implementation experience using a mixed methods approach.

**Methods:**

A five-day training on the WHO IPC CCs was conducted at two reference acute health care facilities in the Democratic Republic of Congo and Burkina Faso. This was accompanied by a three-part mixed-methods evaluation consisting of a: (1) baseline and follow-up survey of participants’ knowledge, attitudes and practices (KAP), (2) qualitative assessment of plenary discussion transcripts and (3) deployment of the WHO IPC assessment framework (IPCAF) tool. Results were analysed descriptively and with a qualitative inductive thematic approach.

**Results:**

Twenty-two and twenty-four participants were trained at each facility, respectively. Baseline and follow-up KAP results suggested increases in knowledge related to the necessity of a dedicated IPC focal person and annual evaluations of IPC training although lack of recognition on the importance of including hospital leadership in IPC training and hand hygiene monitoring recommendations remained. Most participants reported rarely attending IPC meetings or participating in IPC action planning although attitudes shifted towards stronger agreement with the feeling of IPC responsibility and importance of an IPC team. A reocurring theme in plenary discussions was related to limited resources as a barrier to IPC implementation, namely lack of reliable water access. However, participants recognised the importance of IPC improvement efforts such as practical IPC training methods or the use of data to improve quality of care. The facilities’ IPCAF scores reflected a ‘basic/intermediate’ IPC implementation level.

**Conclusions:**

The training and mixed methods evaluation revealed initial IPC implementation experiences that could be used to inform stepwise approaches to facility IPC improvement in resource-limited settings. Implementation strategies should consider both global standards such as the WHO IPC CCs and specific local contexts. The early involvement of all relevant stakeholders and parallel efforts to advocate for sufficient resources and health system infrastructure are critical.

**Supplementary Information:**

The online version contains supplementary material available at 10.1186/s13756-023-01358-1.

## Background

Despite infection prevention and control (IPC) improvement efforts in the last decade, Sub-Saharan African countries continue to face a range of infectious disease threats affecting their population. In June 2021, the Democratic Republic of Congo (DRC) experienced a third wave of severe acute respiratory syndrome coronavirus type 2 (SARS-CoV-2) infections, where the Delta variant (B.1.617.2) was found to be dominant [1, 2]. The Omicron variant was later documented in the country in November, and subsequently, a fourth wave of infections emerged in December 2021 [2]. In the same year, the health system in DRC faced its 12th Ebola virus disease (EVD) outbreak, which began as a resurgence from a survivor of a previous outbreak and had a 50% mortality rate [3]. The 12th outbreak was officially declared over in May, but only five months later, the 13th Ebola outbreak occurred in October 2021 [4]. Similarly, Burkina Faso (BF) was affected by the COVID-19 pandemic, with its two biggest initial waves occurring in December 2020 and 2021 and resulting in a total of 21,128 cases [5]. Furthermore, its central location in west Africa with six border countries makes Burkina Faso a concentrated area of human movement at high-risk for transborder disease transmission. An additional image file shows a map of this movement in more detail (see Additional file 1) [6].

Such challenges demonstrate the need for robust IPC measures that can not only combat infections in emergency outbreak situations, but are established as routine practices and procedures embedded in effective and sustainable IPC programmes at the national and healthcare facility level.

Evidence-based IPC interventions have been shown to prevent more than 50% of health care-associated infections (HAIs), increasing patient and healthcare worker (HCW) safety [7–9]. In 2016, the World Health Organization (WHO) published recommendations for the core components (CC) of IPC programmes [10]. However, in resource-limited settings, where HAI prevalence has been estimated to be 2–3 times more than in settings in Europe and the United States, the implementation of IPC CCs can be challenging for healthcare facilities due to lack of personnel, infrastructure and financial resources [11]. It is essential to determine how IPC guidelines can be effectively implemented in these areas [12]. A recent appraisal from African experts in the Pan African Medical Journal emphasized the contribution of nosocomial COVID-19 infection in the region and IPC programmatic challenges related to weak healthcare systems and infrastructure [13]. Robust evidence on IPC implementation strategies in low-resource settings remains limited, although selected studies have been published in recent years. In 2021, Tomczyk et al. qualitatively assessed IPC implementation themes from a series of interviews conducted with IPC experts from low-resource settings. A range of critical actions were identified that could be taken to achieve the WHO IPC CCs, such as continuous leadership advocacy, initial external technical assistance followed by local guideline adoption, establishment of local IPC career paths and pilots for HAI surveillance and monitoring, audit and feedback among other themes [7].

Our study aimed to add to the evidence base by describing the initial WHO IPC CC implementation experience at two reference hospitals in low-resource settings in the DRC and BF. A training was carried out on the WHO CCs of an IPC programme, and a mixed methods study was conducted to assess healthcare worker (HCW) knowledge, attitudes and practice (KAP), identify context-specific challenges to IPC programme implementation and evaluate the facility level of IPC implementation using the WHO Infection Prevention and Control Assessment Framework (IPCAF) [14].

## Methods

### Study setting

This study takes place in two reference acute health care facilities in Sub-Saharan Africa. Saint Luc Hospital of Kisantu (referred to as ‘Facility A’) is a general reference hospital with 340 beds, serving a population of 190,800 in the Kisantu Health Zone in DRC’s Kongo Central Province in Central Africa The hospital has eight departments (internal medicine, surgery, pediatrics, gynecology, obstetrics, orthopedics, dentistry and ophthalmology) and employs approximately 108 HCW and 60 administrative personnel [15]. Centre University Hospital of Souro Sanou (referred to as ‘Facility B’) is a national referral hospital in Bobo-Dioulasso, BF, with 650 beds, serving several regions with a combined population of over six million. The hospital has six departments (surgery, obstetrics and reproductive medicine, medicine, pediatrics, pharmacy and laboratory) and employs 927 HCWs and 124 administrative staff. Both facilities are partner hospitals in the African Network for improved Diagnostics, Epidemiology and Management of Common Infectious Agents (ANDEMIA), and the study was conducted as part of this partnership [16]. Following discussions with all ANDEMIA network facility leadership during the COVID-19 pandemic response, these two health care facilities were identified as those who expressed the most urgent need for IPC improvement.

**Table 1:** WHO IPC CC training programme participant characteristics in facilities in DRC and BF, 2021–2022^§^

Characteristics	Facility A (*N* = 22)* n (%)	Facility B (*N* = 24)* n (%)
Profession		
Medical Doctor	6 (27.3%)	4 (16.7%)
Nurse	11 (50.0%)	8 (33.3%)
Midwife	1 (4.5%)	1 (4.2%)
Environmental Hygienist	0	5 (20.8%)
Technician (Biologist)	2 (9.1%)	0
Pharmacist	0	2 (8.3%)
Administration	1 (4.5%)	4 (16.7%)
Other	1 (4.5%)	0
Affiliation		
Hospital	14 (63.6%)	19 (79.2%)
Health Zone	4 (18.2%)	0
Central Health Bureau	2 (9.1%)	0
National Health Institute	2 (9.1%)	5 (20.8%)
Additional characteristics		
Member of Hygiene Committee	11 (50.0%)	10 (41.7%)
Reported previous formal IPC training	6/21 (28.6%)	15 (62.5%)
Reported years of work experience (median, IQR)	6.5 years (3–15)	7.0 years (4–12)

¥¥If missing data were present, denominators were specified accordingly within the table (ex: #/*N* (%))

§ Abbreviations: Burkina Faso (BF), Core Component (CC), Democratic Republic of Congo (DRC), Infection Prevention and Control (IPC), Interquartile Range (IQR), World Health Organization (WHO)

**Table 2:** Selected WHO IPC CC training participant responses to knowledge questions in DRC and BF, 2021–2022^§^

Selected knowledge questions	Preferred response [10, 19]	Facility A (*N* = 22)^¥^			Facility B (*N* = 24)^¥^		
		Baseline *n* (%)	Follow-up *n* (%)	*p*-value	Baseline *n* (%)	Follow-up *n* (%)	*p*-value
**CC1**							
There must be at least one trained and dedicated IPC focal person for a minimum of how many beds in the facility?	“at least one professional per 250 beds”	3 (13.6%)*	21 (95.5%)	< 0.001	10/23 (43.5%)*	19 (79.2%)	0.001
**CC2**							
It is essential to monitor the implementation of the IPC guidelines.	“True, regular monitoring of IPC guidelines should be established”	21 (95.5%)	21 (95.5%)	1.000	24 (100%)	24 (100%)	-
**CC3**							
At a minimum, how often should the effectiveness of IPC training be evaluated?	“Establish regular, at least annual, evaluations of the effectiveness of training”	0*	16 (72.8%)	0.001	6 (25.0%)*	13 (54.2%)	0.017
Theoretical training in IPC is more effective than practical training.	“False, use a blended approach to training including…interactive and practical sessions (including simulation and/or bedside training)”	18 (81.8%)*	19 (86.4%)	0.833	23 (95.8%)	22 (91.7%)*	0.564
IPC training should be provided to all front-line clinical staff and environmental/maintenance workers	“True, WHO has identified targets for IPC training: IPC specialists (doctors, nurses), …auxiliary service staff, cleaners, etc”	19 (86.4%)	18 (81.8%)	0.368	22 (91.7%)*	24 (100%)	0.157
IPC training does not have to target senior managers with IPC experience.	“False, WHO has identified targets for IPC training…administrative and managerial staff”	9 (40.9%)	9 (40.9%)	0.607	6/23 (26.1%)	6 (25.5%)	1.000
IPC training and education should be considered for patients and family members	“True, tailored IPC education for patients or family members should be considered to minimize the potential for HAI”	15 (68.2%)	22 (100%)	0.030	22 (91.7%)	22 (91.7%)	1.000
**CC4**							
Hospital administration is not considered a key player that needs feedback on healthcare-associated infection (HAI) surveillance.	“False, surveillance reports should be disseminated …to those at the administration level”	16 (72.7%)*	16 (72.7%)	0.210	23 (95.8%)	23 (95.8%)	0.368
Healthcare-associated infection (HAI) surveillance systems can evaluate the effectiveness of IPC interventions.	“True, surveillance of HAI and antimicrobial resistance (AMR) can…guide IPC strategies and priorities and assess the effectiveness and impact of interventions”	19 (86.7%)*	21 (95.5%)	0.135	23 (95.8%)	24 (100%)	0.317
A system should be in place to assess the quality of surveillance data.	“True, a system for surveillance data quality assessment is of the utmost importance.”	22 (100%)	21 (95.5%)	0.317	23 (95.8%)	24 (100%)	0.317
**CC5**							
Multimodal thinking means that IPC practitioners focus only on single strategies for changing practices.	“False, multimodal thinking means that IPC practitioners do not focus only on single strategies to change practices but rather several integrated elements.”	10 (45.5%)*	15 (68.2%)*	0.160	19 (79.2%)*	20 (83.3%)	0.204
**CC6**							
How often should hand hygiene practices be monitored?	“Regular monitoring should be established”	9 (40.9%)	13 (59.1%)	0.375	8/22 (36.4%)*	6 (25.0%)	0.384
**CC7**							
Staffing should be appropriately distributed according to patient volume.	“True, staffing levels should be adequately assigned according to patient workload.”	17 (77.3%)	18 (81.8%)	0.565	19 (79.2%)*	23 (95.8%)	0.135
Decisions regarding workload, staffing and bed occupancy are the sole responsibility of the IPC Focal Point.	“False, decisions regarding workload, staffing and bed occupancy…also lie with senior managers and directors.”	15 (68.2%)*	17 (77.3%)	0.587	19 (79.2%)*	24 (100%)	0.082
Overcrowding has been recognized as a public health problem that can lead to disease transmission.	“True, overcrowding is also recognized as being a public health issue that can lead to disease transmission.	22 (100%)	22 (100%)	-	20 (83.3%)	22 (91.7%)	0.370
WHO recommends which of the following for bed occupancy standards?	“The importance of not exceeding one patient per bed and ensuring adequate bed locations and space between beds (> 1 m) to reduce the transmission risk and ensure patient safety”	8 (36.4%)	11 (50.0%)	0.314	7/21 (33.3%)*	10/19 (52.6%)	0.174
**CC8**							
Burial in a secure pit is considered an appropriate method of waste disposal in primary and secondary care facilities.	“True, waste should be treated and disposed of safely via autoclaving, incineration, and/or buried in a lined, protected pit”	16 (72.7%)	18 (81.8%)	0.565	10 (41.7%)	15 (62.5%)	0.272
Sufficient and appropriately labelled bins for health care waste segregation should be available within how many meters from the point of generation?	“Sufficient and appropriately labelled bins to allow for health care waste segregation should be available and used (less than 5 m from point of generation)”	2 (9.1%)	18 (81.8%)	0.007	3 (12.5%)*	17/23 (73.9%)	0.008
In a hospital facility, a toilet is needed per how many users?	“A minimum of two functional, improved sanitation facilities that safely contain waste available for outpatient wards should be available and one per 20 beds for inpatient wards”	11 (50.0%)*	21 (95.5%)	0.019	14 (58.3%)*	21 (87.5%)	0.097
**General IPC**							
Standard precautions should only be observed in the event of a COVID-19 or Ebola outbreak.	“False, standard precautions: … must be applied to ALL patients who require health care, by ALL health workers in ALL health settings”	18 (81.8%)	16 (72.7%)	0.368	23 (95.8%)	22 (91.7%)	0.317
Hand hygiene: What are the preferred methods for washing hands if they are visibly soiled?	“Water and soap…ABHR is not a substitute for soap and water for hand hygiene after toileting or when hands are visibly soiled”	17/20 (85.0%)	22 (100%)	0.392	17/18 (94.4%)	19/19 (100%)	0.317

¥If missing data were present, denominators were specified accordingly within the table; comparisons between baseline and follow-up were tested with a paired analysis using the Stuart-Maxwell Marginal homogeneity test (*p*-values)

*More than 5% reported “I don’t know”

§Abbreviations: Alcohol-Based Hand Rub (ABHR), Antimicrobial Resistance (AMR), Burkina Faso (BF), Core Component (CC), Democratic Republic of Congo (DRC), Healthcare-associated Infection (HAI), Infection Prevention and Control (IPC), World Health Organization (WHO)

### Study design

The purpose of this study was to describe the initial WHO IPC CC implementation experience at the selected facilities. Interest in developing an IPC programme was expressed by the facilities and a five-day interactive training programme on the WHO IPC CCs was conducted. Multidisciplinary participants were nominated by hospital leadership as representatives responsible for IPC (e.g. part of the acting hygiene committees or facility leadership teams) across the professional hierarchy. Participation in the training and study was voluntary. The training material was developed based on available WHO guidance by national IPC experts including the input from a global IPC expert [17, 18]. The training programme was delivered by the respective national IPC experts with the engagement of local environmental hygienists. The training was conducted in Facility A in September 2021 and in Facility B in March 2022. These training times were identified by the facilities according to the timing of their COVID-19 pandemic response activities and availability of participants and trainers. In addition, a basic provision of IPC supplies was procured for the facilities to support the initial built environment for IPC. Alongside the conducted training and basic provision of IPC supplies, a three-part mixed methods study was conducted, consisting of: (1) a baseline and follow-up participant KAP survey, (2) a qualitative assessment of plenary discussion transcripts to identify context-specific barriers and facilitators to IPC programme implementation and (3) the guided use of the WHO IPCAF to evaluate the facility level of IPC implementation.

**Table 3:** Selected WHO IPC CC training participant responses to attitude statements in DRC and BF, 2021–2022^§^

Statement	Facility A (*N* = 22)^¥^			Facility B (*N* = 24)^¥^		
	Baseline Median (IQR)	Follow-up Median (IQR)	*p*-value*	Baseline Median (IQR)	Follow-up Median (IQR)	*p*-value*
I can dedicate time to participating in an infection prevention and control (IPC) program.	6.0 (6.0–7.0)	6.0 (6.0–7.0)	0.157	7.0 (6.0–7.0)	7.0 (6.0–7.0)	0.329
I have seen evidence that IPC programmes can control the spread of infection in health care facilities.	6.0 (6.0–7.0)	7.0 (6.0–7.0)	0.056	7.0 (6.5-7.0)	7.0 (7.0–7.0)	0.059
I know the core components of an IPC programme (i.e. World Health Organization guidelines).	5.0 (3.0–6.0)	6.0 (6.0–7.0)	0.001	5.0 (2.5-7.0)	7.0 (7.0–7.0)	< 0.001
Involvement in an IPC programme is one of my responsibilities.	6.0 (6.0–7.0)	7.0 (6.0–7.0)	0.019	7.0 (5.5-7.0)	7.0 (6.0–7.0)	0.033
It is important to use IPC guidelines on specific procedures.	6.0 (6.0–7.0)	6.0 (6.0–7.0)	0.479	7.0 (6.0–7.0)	7.0 (7.0–7.0)	0.025
An IPC programme will protect my own health.	6.5 (6.0–7.0)	6.5 (6.0–7.0)	1.000	7.0 (6.0–7.0)	7.0 (7.0–7.0)	0.008
It is important to my facility to have an active IPC team	7.0 (6.0–7.0)	7.0 (6.0–7.0)	0.412	7.0 (7.0–7.0)	7.0 (7.0–7.0)	0.174
My facility has sufficient funds to support an active IPC programme.	3.0 (2.0–4.0)	5.0 (2.0–5.0)	0.038	4.0 (3.0-5.5)	5.0 (3.5-6.0)	0.450
Senior leadership promotes the formation of an IPC programme at my facility.	6.0 (5.5–6.5)	6.0 (6.0–7.0)	0.375	6.0 (5.0–7.0)	6.0 (5.0–7.0)	0.323
There are no barriers to implementing an IPC programme in my facility.	6.0 (6.0–7.0)	6.0 (5.0–6.0)	0.531	6.0 (5.0-6.5)	3.5 (2.0–5.0)	< 0.001
An IPC programme can function in my facility over a long period of time.	6.0 (6.0–7.0)	6.0 (6.0–7.0)	0.477	6.0 (6.0–7)0.0	6.0 (6.0–7.0)	0.324
An IPC programme will protect the health of patients.	6.5 (6.0–7.0)	7.0 (6.0–7.0)	0.705	7.0 (7.0–7.0)	7.0 (7.0–7.0)	0.180
In my facility, there is adequate access to personal protective equipment (gowns, masks, gloves, eye protection).	5.0 (5.0–6.0)	6.0 (5.0–7.0)	0.047	5.0 (5.0–6.0)	5.0 (5.0–6.0)	0.285

^¥^Median and quartile range according to Likert scale responses (completely disagree = 1, disagree = 2, somewhat disagree = 3, neutral = 4, somewhat agree = 5, agree = 6, completely agree = 7)

*p-value calculated using a paired analysis with the Wilcoxon signed-rank test

§ Abbreviations: Burkina Faso (BF), Core Component (CC), Democratic Republic of Congo (DRC), Infection Prevention and Control (IPC), Interquartile Range (IQR), World Health Organization (WHO)

#### Part one: baseline and follow-up participant KAP survey

A tailored KAP survey on IPC programmes was developed based on the WHO IPC CC and consisted of four sections: participant background characteristics (10 questions), attitudes (13 Likert-scale statements), practices (two yes/no questions, six Likert-scale questions) and knowledge (17 true/false questions, 14 multiple-choice questions, and five open-ended questions). A 7-point Likert scale was used to assess attitudes: completely disagree (1 point), disagree (2 points), slightly disagree (3 points), neutral (4 points), slightly agree (5 points), agree (6 points) and completely agree (7 points). A different Likert scale was used to assess practices, ranging from: never, sometimes, often, always, I don’t know. The knowledge true/false and multiple-choice questions were scored according to the pre-determined correct responses. Using this KAP instrument, a baseline survey was conducted among all training participants on the first day prior to the commencement of the training. Likewise, a follow-up survey with the same instrument and among the same participants was conducted immediately following the conclusion of the training.

**Fig. 1:**
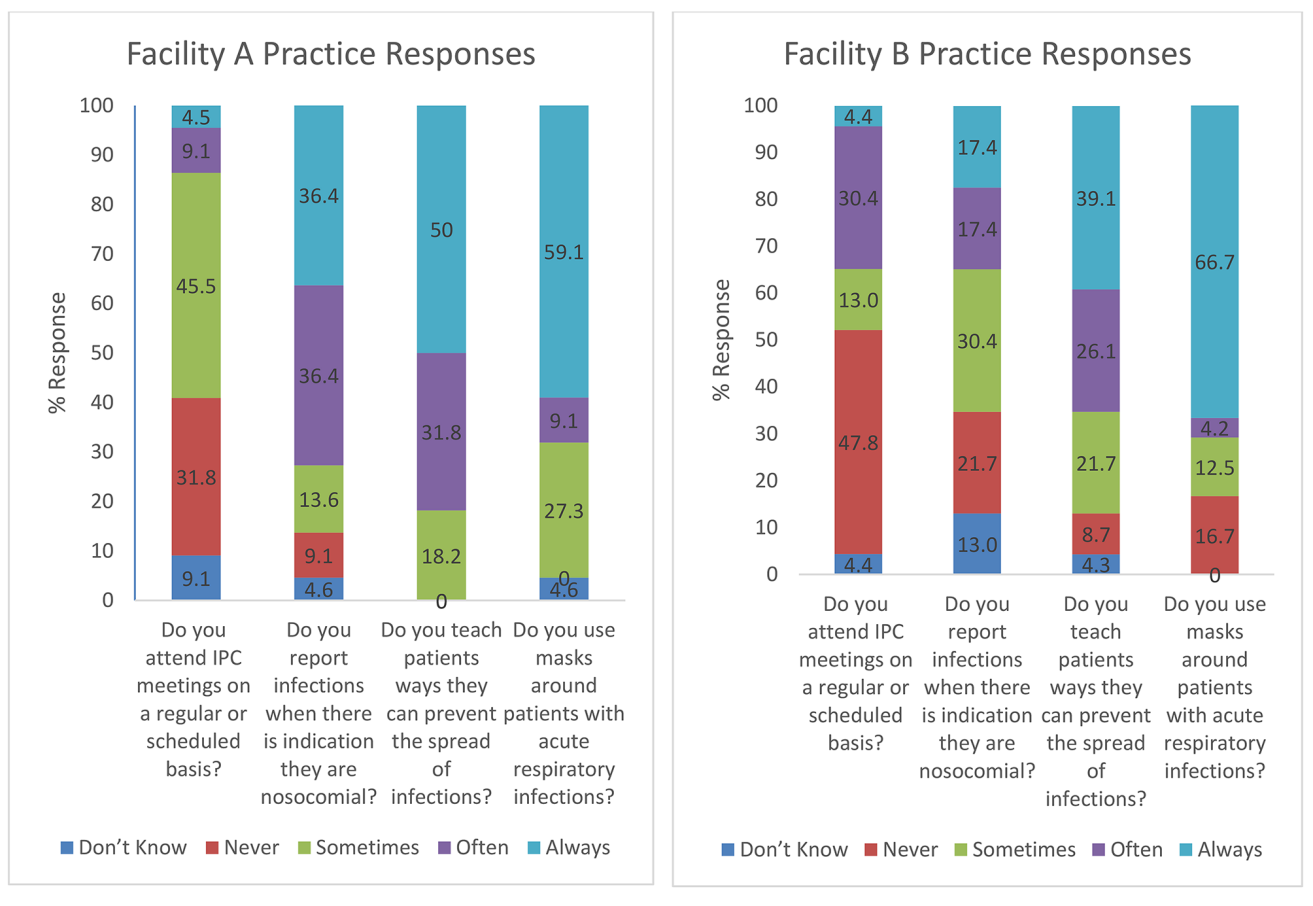
Selected WHO IPC training participant responses to practice questions in DRC and BF, 2021–2022

#### Part two: qualitative assessment of plenary discussions

Interactive plenary discussions were held throughout the training and key points expressed were transcribed for a qualitative assessment of context-specific barriers and facilitators to IPC programme implementation. Daily small group discussions (e.g. consisting of six people) were held for approximately 10–15 min on an assigned topic (e.g. each individual WHO CC). Each small group then nominated a spokesperson to present key conclusions to all training participants in the full plenary for broader discussion.

#### Part three: guided use of IPCAF

The IPCAF is a systematic tool to support the implementation of the WHO CC of IPC programmes at the acute health care facility level. It is a structured closed-formatted questionnaire with an associated scoring system to measure the level of IPC implementation and can act as a progress indicator to facilitate improvement over time [14]. The IPCAF instrument allocates points to each question and a maximum score of 100 points can be achieved for each CC section. An overall score is calculated by adding the total scores of all sections. On the final day of the training, the IPCAF was conducted in the facility. Training participants were divided into four groups and asked to assess two assigned CCs of the ICPAF during a targeted walk-through of the hospital. The completion of the IPCAF was done under the guidance of the IPC expert trainers. Following its completion, the groups were asked to synthesize their findings in a plenary presentation and results were further discussed in the full group.

**Fig. 2:**
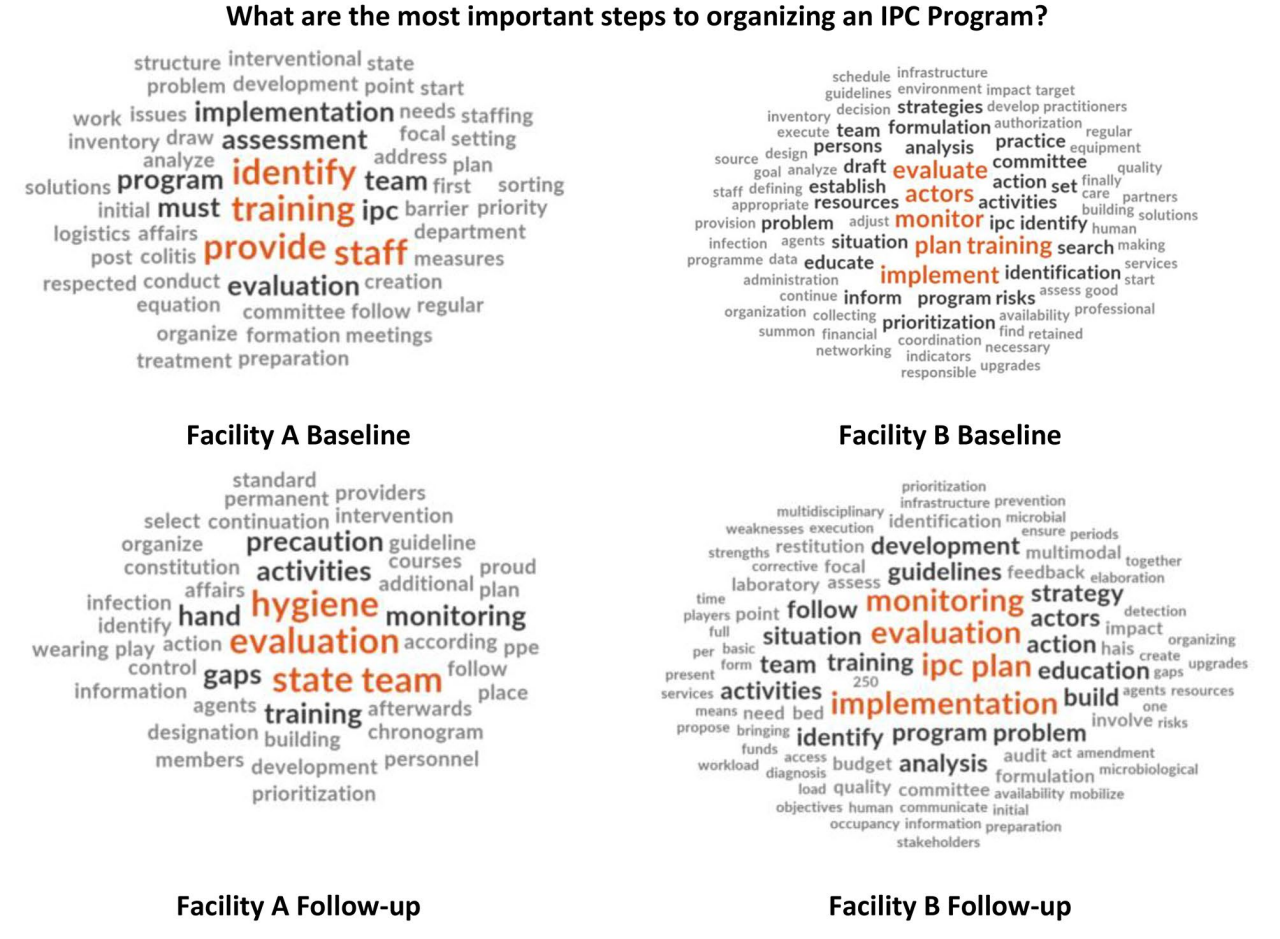
Word cloud comparison of reported IPC programme organization steps between baseline and follow-up per facility

### Statistical analysis

For the participant KAP survey, frequencies and proportions of categorical response proportions were summarized and baseline and follow-up results were compared with a paired analysis using the Stuart-Maxwell Marginal homogeneity test. Median and inter-quartile (IQR) estimates were summarized for the Likert-scale responses to attitude statements, and baseline and follow-up responses were compared with a paired analysis using the Wilcoxon signed-rank test. Baseline practices were described as proportions and histograms, follow-up practice responses were not analyzed because enough time had not passed for changes to practices. Key feedback points from plenary discussions and written responses to the open-ended knowledge questions were analyzed using a qualitative, inductive thematic analysis in which responses were coded first according to WHO IPC CC and then emerging themes for each CC were identified. Themes that emerged more than once were considered to be ‘reoccurring’. Responses to selected open-ended questions were also analyzed for word frequency using word cloud queries. The IPCAF scoring results were analyzed using descriptive statistics. Stata Version 17.0, Nvivo 1.5.2 and Excel were used for analyses.

**Table 4:** Reoccurring themes (*n* ≥ 2) from selected open-ended KAP responses among participants in DRC and BF, 2021–2022^§^

Baseline themes	N*	Follow-up themes	N*
**What is the difference between an IPC team and committee?**			
The larger IPC team makes decisions and the committee is operational.	19	The larger, heterogenous IPC committee makes decisions, and the smaller, homogenous team is operational.	23
The IPC committee makes decisions, and the IPC team is operational.	3	The larger team monitors hygiene activities.	2
**What is the most effective way to train health care workers in the prevention of hospital-acquired infections (HAI)?**			
Practical and/or theoretical training approaches, ideally with context-specific content, should be used.	15	Practical and/or theoretical training approaches should be used.	18
Awareness should be raised through information dissemination (i.e. illustrating importance of measures, risks, responsibilities).	5	Awareness should be raised through information dissemination (i.e. illustrating importance of measures, risks, responsibilities).	7
The facility or the department should to hold follow-up or on demand trainings.	3	It should be ensured that everyone receives training, ideally on an annual basis related to standard and complementary precautions.	5
Training should be done through participatory methods.	3	Participative training or process integration from the beginning of the action should be done.	2
Training should be conducted routinely or in staff meetings.	3	Training should involve HCWs in the whole process of the IPC program.	2
**How can you use healthcare-associated infection surveillance data?**			
Data can be used to improve IPC measures and quality of care.	9	Data can be used to evaluate and improve IPC programmes and quality of care.	13
Data can be used to give feedback and raise awareness for behavioral change.	9	Data can be used to provide feedback for behavioral change, training and decision making.	6
Data can be used to assess and evaluate effectiveness of IPC interventions.	6	Data can be used to guide IPC implementation.	2
Data can be used to provide feedback that can inform decision-making and trainings.	4	Quality assurance indicators can be used.	2
Data can only be used if you have the correct collection tools.	4	Data can be used to reduce costs and advocate for leadership support of IPC program implementation.	2
Indicator can be used to monitor hygiene or quality of care.	3		

*N = number of times themes were coded or identified across participant open-ended KAP responses

§Abbreviations: Burkina Faso (BF), Democratic Republic of Congo (DRC), Healthcare-associated Infection (HAI), Healthcare Worker (HCW), Infection Prevention and Control (IPC), Knowledge, Attitude and Practice (KAP)

#### Ethics approval and consent to participate

The ANDEMIA Project is currently operating in the Democratic Republic of Congo under the ethical approval granted by the Ethics Committee of the University of Kinshasa Deliberation N^o^ ESP/CE/042/2017, in Burkina Faso under the ethical approval granted by the Ethics Committee by the Burkina Faso Ministry of Health Deliberation N^o^ 2017-5-057, and the German Charité Medical University EA2/230/17.

**Table 5:** Reoccurring themes (*n* ≥ 2): IPC programme challenges and facilitators in discussions in DRC and BF, 2021–2022^§^

Themes	N*
CC 1: IPC Programme	
Personnel attitudes are a barrier (including misperceptions or lack of awareness and commitment)	7
Limited resources are a barrier (including human resources)	4
Organizational issues and unclear responsibilities are a barrier	4
Ministry of Health guideline alignment**	1
CC 2: IPC Guidelines	
Insufficient available protocols and procedures and resulting implementation are barriers	2
Insufficient involvement of and communication between actors are barriers	2
CC 3: IPC Education and Training	
NA (No reoccurring themes identified)	-
CC 4: HAI Surveillance	
Limited resources for surveillance are a barrier	4
Insufficient data collection and reporting are barriers	2
CC 5 Multimodal strategies	
Attitudes towards and knowledge of multimodal strategies are barriers	3
Limited resources for multimodal strategies are a barrier	2
CC 6 Monitoring, audits of IPC practices and feedback	
Lack of training, audit programmes and resulting feedback	3
CC 7 Workload, staffing and bed occupancy	
Organizational issues are a barrier	4
Limited resources for staffing and bed occupancy are a barrier	2
CC 8 Built environment, materials and equipment for IPC at the facility level	
Limited resources for built environment are a barrier	17
Reliable access to water is essential	5

**this theme only occurred once, but was considered to be of importance and therefor included in the table.

*N = number of times themes were coded or identified across participant open-ended KAP responses

§Abbreviations: Burkina Faso (BF), Democratic Republic of Congo (DRC), Healthcare-associated Infection (HAI), Infection Prevention and Control (IPC), Knowledge, Attitude and Practice (KAP)

**Fig. 3:**
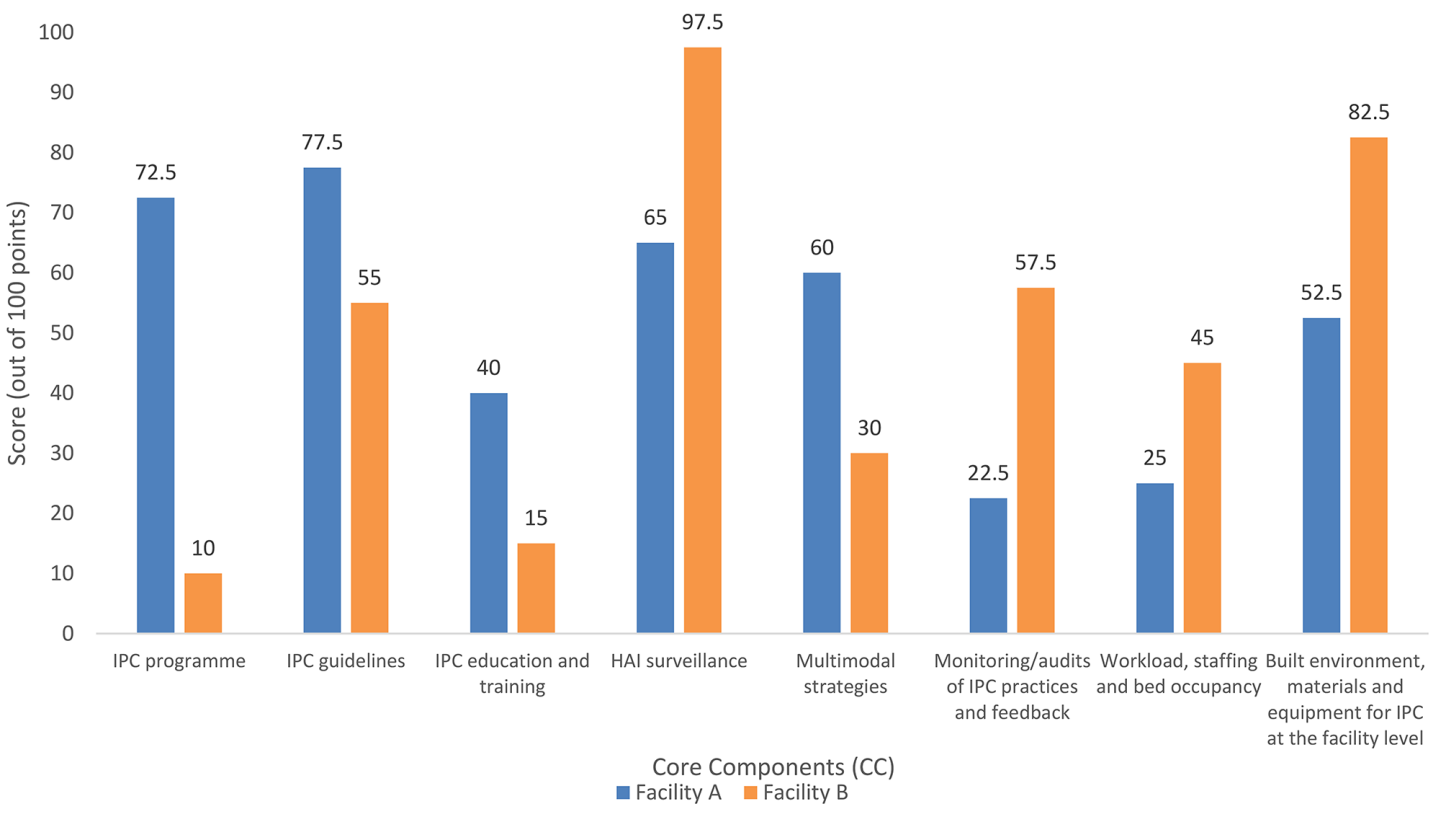
IPCAF results from facilities in DRC and BF, 2021–2022

## Results

### Participant characteristics

A total of 22 and 24 individuals participated in separate five-day WHO IPC CC training programmes in Facility A (September 2021) and Facility B (March 2022), respectively. The participants were predominately HCWs, with the largest professional groups being medical doctors and nurses (see Table 1 below). Approximately half of the training participant groups were members of the respective hygiene committees for each facility. In Facility A, it was also considered necessary to include external participants from the affiliated Health Zone Departments and the Central Health Bureau. Alongside the training, the facilities prioritized basic IPC supplies which were procured for the hospital, including personal protective equipment (PPE) as well as consumables for hand hygiene and waste management.

### Knowledge, attitudes and practices (KAP) survey

Participant responses to selected knowledge questions in the KAP survey are shown in Table 2. Overall, participants demonstrated a high understanding of questions related to standard precautions, importance of HAI surveillance, practical IPC training, monitoring the implementation of IPC guidelines and standards for staffing and bed occupancy at both time points. From baseline to follow-up, participants in both facilities showed a significant increase in understanding of questions related to the necessity of a dedicated IPC focal person, at least annual evaluations of IPC training, healthcare waste segregation standards (*p* < 0.01) as well as a modest increase in the understanding of toilet facility standards. However, gaps at both the baseline and follow-up timepoints included lack of recognition on the importance of including senior hospital leadership in IPC training and the necessity to monitor hand hygiene compliance.

Participant responses to attitude statements are shown in Table 3 below. High agreement with the perception that one can dedicate time to an IPC programme was seen at both timepoints. There was a significant increase in agreement with the feeling of responsibility to IPC and understanding of the IPC core components from baseline to follow-up (*p*-value < 0.04). At Facility A, significantly more participants from baseline to follow-up agreed with the attitude that sufficient funds for IPC were available (*p*-value < 0.04). However, participants from Facility B reported a stronger feeling of barriers to IPC programme implementation from baseline to follow-up (*p*-value < 0.001).

Participant responses to practice questions at baseline are reported in Fig. 1. A majority of participants at both facilities reported never or only sometimes attending regular IPC meetings and few reported ever being part of a process to draft an action plan to address identified IPC needs (9.1% Facility A, 37.5% Facility B; not shown in Figure below). However, a majority reported often or always adhering to practices such as teaching patients about IPC and using masks when caring for patients with acute respiratory infections.

In addition, the open-ended KAP question “What are the most important steps to organizing an IPC program?” was analyzed using a word cloud to show frequency of responses (see Fig. 2 below). From baseline to follow-up, facility responses appeared to show a shift in participants stressing individual training to emphasizing the concept of an IPC team as well as evaluation, monitoring and implementation. A word cloud analysis was also conducted for the question ‘Once IPC guidelines have been developed, what steps should be taken to ensure their implementation at the facility?’ and can be viewed as an additional file (see Additional file 2).

Reoccurring themes identified in responses to the three-remaining open-ended KAP questions were analyzed using a thematic analysis (see Table 4 below). Most frequent reoccurring themes included statements related to the role of the IPC committee for decision-making compared to the operational role of the IPC team as well as the need for effective IPC trainings to consist of both practical and theoretical components. There were also reocurring themes related to the use of HAI data for improving quality of care, evaluating IPC programmes, or providing feedback to inspire behavioural change.

All qualitative themes can be viewed as an additional file (see Additional file 3).

### Plenary interactive discussions

The reoccurring themes of IPC programme challenges from the interactive plenary discussion sessions were identified according to CC in Table 5. Limited resources as a key barrier emerged as a theme across all CCs. Resources mentioned ranged from material and financial to human resources, and related misconceptions such as the use of handwashing with ash when there was a shortage of water or soap, decontamination or sterilization with inappropriate substances or the multiuse of single-use items were noted. Others expressed concerns with having a 100% dedicated person for IPC such as how to employ a new person in general and how to take on hospital staff and exempt them from clinical charges despite other needs in the hospital. Another dominant theme was that personnel attitudes were a major barrier to IPC programmes, including misperceptions and lack of awareness and commitment. Some participants expressed that “IPC is still considered a new concept that resulted from various epidemics, so it is not needed in non-epidemic times.” Others expressed that there is an insufficient commitment from health care facility management and a lack of responsibility among staff and users regarding compliance with IPC measures. The dominant theme of ‘Water is essential’ also emerged in the context of CC 8, with statements such as “water is life” and detailed discussions on available water sources and uses. In Facility B, it was estimated that 143 L of water are needed per hospitalized patient (per 24-hour day). Participants also suggested potential solutions and facilitators. One proposed plenary solution was to align Ministry of Health guidelines (CC1 theme ‘Ministry of Health alignment’) hygiene committee guidelines with respective facility IPC committees. Furthermore, it was discussed that conveying the HCW and patient benefits of IPC might combat misperceptions of IPC importance.

### Facility IPCAF evaluations

The overall IPCAF score at Facility A (392.5/800 points) corresponded to a ‘Basic’ IPC level: “Some aspects of the IPC core components are in place, but not sufficiently implemented. Further improvement is required” (Fig. 3). The lowest ranked component was CC1 IPC programmes (10/100), and the highest ranked component was CC4 Healthcare-associated infection (HAI) surveillance (97.5/100). The IPCAF score at Facility B (415/800 points) corresponded to an ‘Intermediate’ IPC level: “Most aspects of the IPC core components are appropriately implemented. The facility should continue to improve the scope and quality of implementation and focus on the development of long-term plans to sustain and further promote the existing IPC programme activities.” [14]. The lowest ranked component was CC6 Monitoring, audits of IPC practices and feedback (22.5/100) and the highest ranked component was CC2 IPC guidelines (77.5/100).

## Discussion

We evaluated the initial WHO IPC CC implementation experience at two reference hospitals in the DRC and BF. Overall, these facilities demonstrated a basic to intermediate IPC baseline level, using the WHO IPCAF tool. This level of IPC implementation is comparable to the findings of other countries in low-income settings and within the African region according to a 2022 WHO global IPC survey in acute healthcare facilities [20–22]. Using mixed evaluation methods during and following a training on the WHO IPC CCs at the two reference facilities, a range of IPC implementation experiences and challenges were identified that could be used to inform future IPC improvement strategies.

Some elements of an IPC programme (i.e. WHO IPC CC1) were reported in place at the facilities according to the WHO IPCAF tool. However, the KAP survey and assessment of plenary discussions revealed perceptions and practices affecting the effectiveness of IPC programme implementation at the facilities. Most training participants reported rarely attending regular IPC meetings and only a few participants reported involvement in a process to draft an IPC programme action plan. Following the training, participant responses shifted from stressing the need for more individual training to emphasizing the concept of an IPC team, responsibility for ensuring IPC and implementation elements such as evaluation and monitoring. Although training participants also demonstrated an increased recognition that healthcare facilities should have a dedicated IPC focal point, concerns were expressed regarding the practicalities of hiring a dedicated IPC focal person when additional staff are needed throughout the facilities to meet ongoing gaps in clinical services and patient management. Participants also highlighted a lack of commitment from hospital leadership as a potential barrier to IPC programme implementation. Interestingly, participants, however, did not believe that senior staff needed to be included in IPC training. This could be related to local hierarchical structures and practices, but inclusion of leadership in IPC training can be important to increase IPC awareness and buy-in. Similar thematic issues were also discussed in a qualitative study on IPC implementation in low-resource settings from Tomczyk et al., and suggestions were made to begin with a stepwise approach, i.e. “start with a small group of committed staff”, “ maintain continuous advocacy…with the inclusion of IPC in routine meetings” [7]. Such IPC champions and awareness-raising could support a paradigm shift from IPC as a “concept to only be used during epidemics” to a mindset that a robust IPC programme should be functioning at all times within a healthcare facility to ensure quality of care and patient safety. However, limited resources were raised as a key barrier throughout the training and evaluation, and global, regional and national health system initiatives are needed in parallel to ensure sufficient human resources and infrastructure for universal health coverage [23, 24]. One proposed plenary solution to IPC programme barriers, was to align Ministry of Health hygiene committee guidelines with respective facility IPC committees. The alignment would make it easier to access national support and manage limited human resources. Furthermore, it was discussed that conveying the HCW and patient benefits of IPC might combat misperceptions of IPC importance. Evidence on benefits might elevate perceived importance of IPC measures and therefor improve HCW ownership and compliance.

Participants reported strong agreement with the importance of IPC guidelines (i.e. WHO IPC CC2) and training (i.e. WHO IPC CC3) including monitoring their implementation. However, low IPCAF facility scores were particularly seen for IPC education and training, and reoccurring themes in discussions emphasized the need for improved communication mechanisms and involvement of all actors throughout the implementation process as well as greater recognition of practical or bed-side training approaches to operationalize the implementation of protocols and procedures. In another study at a tertiary care facility in Canada, HCWs also reported that they need more effective IPC communication and recommended a monthly emailed report of less than two pages covering outbreaks, infection rate comparisons (to other hospitals) and general IPC facts [25]. The US Centers for Disease Control and Prevention also issued IPC communication and collaboration recommendations such as fostering collaboration by engaging IPC actors (such as health service leadership and staff) in development of IPC decisions and actions [26]. Greater recognition of active training approaches aligns with WHO recommendations on participatory and bedside simulation strategies [10]. Participants from both facilities also showed a significant increase in knowledge that training and education can include patients and family members. HCWs have been shown to be hesitant to include this group in IPC measures despite WHO recommendations [27, 28].

A high IPCAF score was seen for HAI surveillance (i.e. WHO IPC CC4), substantially higher than comparable facilities in the WHO IPC global survey [20]. This scoring may be biased due to the lack of participant understanding related to what constitutes HAI surveillance due to the lack of training on HAI surveillance standards and requirements. Qualitative participant responses showed that participants understood the value of data as indicators for quality of care and behavioral change, but limited resources and insufficient data collection and reporting systems were cited as ongoing barriers. Studies on HAI surveillance initiatives in lower-middle income hospitals recommend initially focusing a step-wise implementation in select units, such as intensive care, developing protocols that can consistently be used in the local context and using resulting data to emphasize the importance of IPC programmes for continued stakeholder motivation [29–31].

A modest proportion of participants showed an understanding of multimodal IPC strategies (i.e. WHO IPC CC5) throughout the training. However, the term “multimodal strategies” still appears to be a new concept in settings with a basic level of IPC implementation. Although some educational materials have been developed such as infographics by WHO, ongoing and improved communication approaches are needed to introduce and operationalize the concept of multimodal strategies [14].

Participants reported monitoring (i.e. WHO IPC CC6) as an important step in organizing an IPC programme, and the use of feedback (i.e. from monitoring or observation) to facilitate behaviour change was a reoccurring theme in plenary discussions. This reflects the WHO recommendations that monitoring and feedback are essential ways to support behaviour and system change [32]. However, fewer participants demonstrated an understanding of the specific recommendation to routinely monitoring hand hygiene compliance. This could be an effective starting point to operationalize thekey IPC indicators for monitoring, audit and feedback as suggested by Tomczyk et al. [7].

Participants also demonstrated an understanding of the importance of staffing, workload, bed occupancy (i.e. WHO IPC CC7) and sanitation and waste management (i.e. WHO IPC CC8) standards. Adherence to selected precautions such as the use of masks when caring for patients with acute respiratory infections was noted. However, limited resources were again a reoccurring theme for this CC. IPC training in low-resource settings should discuss appropriate low-cost alternatives that still meet minimum standards to avoid potentially harmful reported practices such as hand washing with ash, decontaminating or sterilizing with inappropriate substances or multiuse of single-usage items [33, 34]. Water availability was also heavily discussed with multiple participants emphasizing “Water is Life”. Practical stepwise implementation tools such as the WHO practical manual for improving IPC at the health care facility level [19] and WASH FIT could offer guidance on finding stepwise, low-cost alternatives that still meet IPC standards. The WASH FIT guideline acknowledges that certain actions such as installing a water supply may not be feasible and recommends small actions that can instigate change such as appealing to district authorities for improvement [35].

### Limitations

The mixed methods evaluation utilized to describe and assess the initial WHO IPC CC implementation experience at the reference hospitals in the DRC and BF had limitations that should be considered. Study participation was voluntary and facility stakeholders were included based on their expressed interest in IPC. Thus, it is possible that results of this study may reflect findings where there is a greater than average interest in IPC. The KAP survey was self-administered and responses may have been affected by social-desirability bias or misinterpreted despite initial instructions and guidance upon dissemination. Furthermore, the follow-up survey timepoint was administered directly after the training and additional follow-up will be needed to understand long-term effects. Open-ended questions and plenary discussions were inductively coded and thematically compared, but the coding process may have been biased by the researcher’s subjectivity. Despite guidance provided during the IPCAF administration, social-desirability bias may have also affected the type of responses given.

## Conclusion

The mixed methods employed to evaluate the initial WHO IPC CC implementation experience at the reference hospitals in the DRC and BF revealed a range of implementation experiences, barriers and facilitators that could be used to inform stepwise approaches to the implementation of the WHO IPC CC in low-resource settings. Implementation strategies should consider both IPC standards such as the WHO IPC minimum requirements [10] as well as the specific local context affecting implementation. The early involvement of all relevant stakeholders including health care facility leadership and decision-makers and health care personnel contributing to current or future IPC teams and committees is critical to ensure sufficient support and an effective and sustainable process. Interactive training approaches with mixed evaluation methods and practical tools such as the WHO IPCAF can contribute to improved outcomes and action planning. Communication of benefits for patients and HCWs may improve IPC programme perceptions and compliance. In parallel, ongoing advocacy for health system changes will also be needed to enable sufficient human and material resources for IPC and quality.

### Electronic supplementary material

Below is the link to the electronic supplementary material.

Supplementary Material 1

Supplementary Material 2

Supplementary Material 3

## Data Availability

All data and materials are accessible in the supplementary information.

## Abbreviations

BF: Burkina Faso
CC: Core components
DRC: Democratic Republic of Congo
EVD: Ebola virus disease
HAI: Health care-associated infections
HCW: Healthcare worker
IPC: Infection Prevention and Control
IPCAF: Infection Prevention and Control Assessment Framework
IQR: Interquartile Range
KAP: Knowledge, Attitudes and Practice
INRB: National Institute of Biomedical Research
SARS: CoV-2-Respiratory Syndrome Coronavirus Type 2
WHO: World Health Organization \
