# Supplementary material for: Implementation of the WHO core components of an infection prevention and control programme in two sub-saharan African acute health-care facilities: a mixed methods study
Source: Antimicrob Resist Infect Control. 2024 Jan 15;13:4. doi: 10.1186/s13756-023-01358-1 (PMC10789048; doi:10.1186/s13756-023-01358-1)
Supplement: Supplementary file 1 — Supplementary Material 1 [file 13756_2023_1358_MOESM1_ESM.docx]

Additional File 2. Word Cloud comparison for responses on IPC guideline implementation steps in each facility.

| **Once IPC guidelines have been developed, what steps should be taken to ensure their implementation at the facility?** | |
| --- | --- |
| 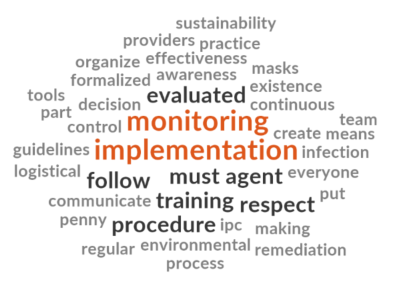 | 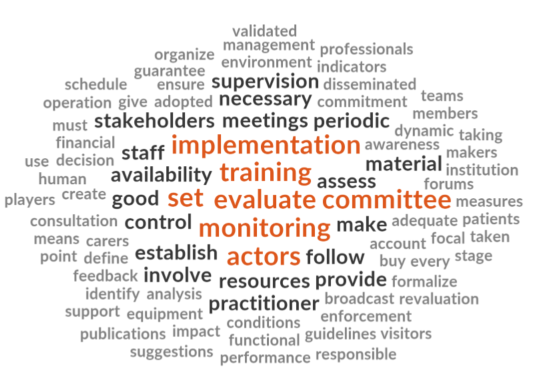 |
| **Facility A Baseline** | **Facility B Baseline** |
| 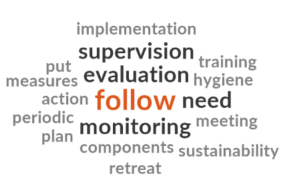 | 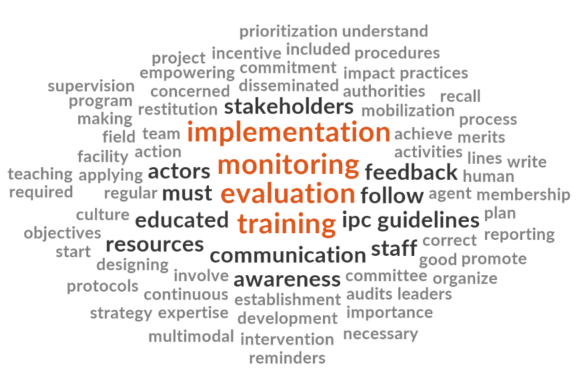 |
| **Facility A Follow-up** | **Facility B Follow-up** |
