# Supplementary material for: Implementation of the WHO core components of an infection prevention and control programme in two sub-saharan African acute health-care facilities: a mixed methods study
Source: Antimicrob Resist Infect Control. 2024 Jan 15;13:4. doi: 10.1186/s13756-023-01358-1 (PMC10789048; doi:10.1186/s13756-023-01358-1)
Supplement: Supplementary file 2 — Supplementary Material 2 [file 13756_2023_1358_MOESM2_ESM.docx]

All qualitative themes can be viewed as an additional file (see Additional file 3).

| Name | Files | References |
| --- | --- | --- |
| Bq1_CHUSS_What are the important steps in organizing an IPC program at a facility | 18 | 18 |
| Bq1_DRC_What are the important steps in organizing an IPC program at a facility | 13 | 13 |
| Bq2_CHUSS_What is the difference between an IPC team and an IPC committee | 17 | 17 |
| Committee is comprised ofseveral teams | 1 | 1 |
| Don't Know | 2 | 2 |
| Each department has a team | 1 | 1 |
| Team is homogenous and Committee heterogenous | 2 | 2 |
| The committee makes decisions and evaluates. The team acts. | 11 | 11 |
| the team is part of the committee | 2 | 2 |
| Bq2_DRC_What is the difference between an IPC team and an IPC committee | 11 | 11 |
| materials | 2 | 2 |
| The committee and team are the same | 1 | 1 |
| The committee and team have different responsibilities (unspecified) | 2 | 2 |
| the committee makes decisions | 1 | 1 |
| the committee makes decisions and coordinates and the team acts | 3 | 3 |
| the team includes the committee | 1 | 1 |
| the team makes decisions and the committee acts | 1 | 1 |
| The team supervises the smaller, subgroup committee | 1 | 1 |
| Bq3_CHUSS_Once IPC guidelines have been developed, what steps should be taken to ensure their implementation at the facility | 20 | 20 |
| Bq3_DRC_Once IPC guidelines have been developed, what steps should be taken to ensure their implementation at the facility (2) | 12 | 12 |
| Bq4_CHUSS_What is the most effective way to train health care workers in the prevention of hospital-acquired infections | 19 | 19 |
| Bq4_DRC_What is the most effective way to train health care workers in the prevention of hospital-acquired infections (2) | 13 | 13 |
| Bq5_CHUSS_How can you use hospital-acquired infection surveillance data (2) | 19 | 19 |
| Bq5_DRC_How can you use hospital-acquired infection surveillance data | 13 | 13 |
| CC5 Multimodal Strategies | 1 | 4 |
| FUq1_CHUSS_What are the important steps in organizing an IPC program at a facility | 22 | 24 |
| FUq1_DRC_What are the important steps in organizing an IPC program at a facility (2) | 13 | 13 |
| FUq2_CHUSS_What is the difference between an IPC team and an IPC committee | 21 | 21 |
| Team is homogenous and Committee heterogenous | 4 | 4 |
| The committee includes the team | 2 | 2 |
| the committee is composed of multiple teams | 1 | 1 |
| The committee makes decisions and evaluates. The team acts. | 14 | 14 |
| the team is larger than the smaller committee | 1 | 1 |
| the team monitors hygiene activites | 1 | 1 |
| FUq2_DRC_What is the difference between an IPC team and an IPC committee (2) | 6 | 6 |
| follow-up content | 1 | 1 |
| The committee includes the team | 1 | 1 |
| The committee makes decisions and evaluates. The team acts. | 2 | 2 |
| the perennial | 1 | 1 |
| the team is global, the committee only has a few representatives | 1 | 1 |
| FUq3_CHUSS_Once IPC guidelines have been developed, what steps should be taken to ensure their implementation at the facility | 22 | 22 |
| FUq3_DRC_Once IPC guidelines have been developed, what steps should be taken to ensure their implementation at the facility (2) | 9 | 9 |
| FUq4_CHUSS_What is the most effective way to train health care workers in the prevention of hospital-acquired infections | 24 | 24 |
| FUq4_DRC_What is the most effective way to train health care workers in the prevention of hospital-acquired infections (2) | 10 | 10 |
| FUq5_CHUSS_How can you use hospital-acquired infection surveillance data | 24 | 24 |
| FUq5_DRC_How can you use hospital-acquired infection surveillance data (2) | 10 | 10 |

Codes\\Codes

| Name | Files | References | |  |
| --- | --- | --- | --- | --- |
| Bq1_CHUSS_What are the important steps in organizing an IPC program at a facility | 18 | 18 | |  |
| Bq1_DRC_What are the important steps in organizing an IPC program at a facility | 13 | 13 | |  |
| Bq2_CHUSS_What is the difference between an IPC team and an IPC committee | 17 | 17 | |  |
| Bq2_DRC_What is the difference between an IPC team and an IPC committee | 11 | 11 | |  |
| Bq3_CHUSS_Once IPC guidelines have been developed, what steps should be taken to ensure their implementation at the facility | 19 | 19 | |  |
| Bq3_DRC_Once IPC guidelines have been developed, what steps should be taken to ensure their implementation at the facility (2) | 13 | 13 | |  |
| Bq4_CHUSS_What is the most effective way to train health care workers in the prevention of hospital-acquired infections | 20 | | 20 | |
| Bq4_DRC_What is the most effective way to train health care workers in the prevention of hospital-acquired infections (2) | 13 | | 13 | |
| Bq5_CHUSS_How can you use hospital-acquired infection surveillance data (2) | 19 | | 19 | |
| Bq5_DRC_How can you use hospital-acquired infection surveillance data | 13 | | 13 | |
| FUq1_CHUSS_What are the important steps in organizing an IPC program at a facility | 22 | | 24 | |
| FUq1_DRC_What are the important steps in organizing an IPC program at a facility (2) | 13 | | 13 | |
| FUq2_CHUSS_What is the difference between an IPC team and an IPC committee | 21 | | 21 | |
| FUq2_DRC_What is the difference between an IPC team and an IPC committee (2) | 6 | | 6 | |
| FUq3_CHUSS_Once IPC guidelines have been developed, what steps should be taken to ensure their implementation at the facility | 22 | | 22 | |
| FUq3_DRC_Once IPC guidelines have been developed, what steps should be taken to ensure their implementation at the facility (2) | 9 | | 9 | |
| FUq4_CHUSS_What is the most effective way to train health care workers in the prevention of hospital-acquired infections | 24 | | 24 | |
| FUq4_DRC_What is the most effective way to train health care workers in the prevention of hospital-acquired infections (2) | 10 | | 10 | |
| FUq5_CHUSS_How can you use hospital-acquired infection surveillance data | 24 | | 24 | |
| FUq5_DRC_How can you use hospital-acquired infection surveillance data (2) | 10 | | 10 | |

Codes\\Open-Ended Questions

Codes\\Open-Ended Questions\\Q2 Sub-Themes

| Name | Description | | | Files | References |
| --- | --- | --- | --- | --- | --- |
| Bq2_CHUSS_What is the difference between an IPC team and an IPC committee | 17 | 17 |  |  |  |
| Committee is comprised of several teams | 1 | 1 |  |  |  |
| Don't Know | 2 | 2 |  |  |  |
| Each department has a team | 1 | 1 |  |  |  |
| Team is homogenous and Committee heterogenous | 2 | 2 |  |  |  |
| The committee makes decisions and evaluates. The team acts. | 11 | 11 |  |  |  |
| the team is part of the committee | 2 | 2 |  |  |  |
| Bq2_DRC_What is the difference between an IPC team and an IPC committee | 11 | 11 |  |  |  |
| materials | 2 | 2 |  |  |  |
| The committee and team are the same | 1 | 1 |  |  |  |
| The committee and team have different responsibilities (unspecified) | 2 | 2 |  |  |  |
| the committee makes decisions | 1 | 1 |  |  |  |
| the committee makes decisions and coordinates and the team acts | 3 | 3 |  |  |  |
| the team includes the committee | 1 | 1 |  |  |  |
| the team makes decisions and the committee acts | 1 | 1 |  |  |  |
| The team supervises the smaller, subgroup committee | 1 | 1 |  |  |  |
| FUq2_CHUSS_What is the difference between an IPC team and an IPC committee | 21 | 21 |  |  |  |
| Team is homogenous and Committee heterogenous | 4 | 4 |  |  |  |
| The committee includes the team | 2 | 2 |  |  |  |
| the committee is composed of multiple teams | 1 | 1 |  |  |  |
| The committee makes decisions and evaluates. The team acts. | 14 | 14 |  |  |  |
| the team is larger than the smaller committee | 1 | 1 |  |  |  |
| the team monitors hygiene activites | 1 | 1 |  |  |  |
| FUq2_DRC_What is the difference between an IPC team and an IPC committee (2) | 6 | 6 |  |  |  |
| follow-up content | 1 | 1 |  |  |  |
| The committee includes the team | 1 | 1 |  |  |  |
| The committee makes decisions and evaluates. The team acts. | 2 | 2 |  |  |  |
| the perennial | 1 | 1 |  |  |  |
| the team is global, the committee only has a few representatives | 1 | 1 |  |  |  |

Codes\\Open-Ended Questions\\Q2 Sub-Themes\\Q2 themes

| Name | Files | References |
| --- | --- | --- |
| Bq2_CHUSS_What is the difference between an IPC team and an IPC committee | 17 | 17 |
| Committee is comprised of several teams | 1 | 1 |
| Each department has a team | 1 | 1 |
| The larger, heterogenous committee makes decisions, and the smaller, homogenous team is operational | 0 | 0 |
| Team is homogenous and Committee heterogenous | 2 | 2 |
| The committee makes decisions and evaluates. The team acts. | 11 | 11 |
| the team is part of the committee | 2 | 2 |
| Bq2_DRC_What is the difference between an IPC team and an IPC committee | 11 | 11 |
| The committee and team are the same | 1 | 1 |
| The committee makes decisions, and the team is operational | 0 | 0 |
| the committee makes decisions | 1 | 1 |
| the committee makes decisions and coordinates and the team acts | 3 | 3 |
| the larger team makes decisions and the committee is operational | 0 | 0 |
| the team includes the committee | 1 | 1 |
| the team makes decisions and the committee acts | 1 | 1 |
| The team supervises the smaller, subgroup committee | 1 | 1 |
| FUq2_CHUSS_What is the difference between an IPC team and an IPC committee | 21 | 21 |
| the committee is composed of multiple teams | 1 | 1 |
| the larger team monitors hygiene activities | 0 | 0 |
| the team is larger than the smaller committee | 1 | 1 |
| the team monitors hygiene activites | 1 | 1 |
| The larger, heterogenous committee makes decisions, and the smaller, homogenous team is operational | 0 | 0 |
| Team is homogenous and Committee heterogenous | 4 | 4 |
| The committee includes the team | 2 | 2 |
| The committee makes decisions and evaluates. The team acts. | 14 | 14 |
| FUq2_DRC_What is the difference between an IPC team and an IPC committee (2) | 6 | 6 |
| The larger committee makes decisions, and the smaller team is operational | 0 | 0 |
| The committee includes the team | 1 | 1 |
| The committee makes decisions and evaluates. The team acts. | 2 | 2 |
| the team is global, the committee only has a few representatives | 1 | 1 |

Codes\\Open-Ended Questions\\Q4 Sub-Themes

| Name | Files | References |
| --- | --- | --- |
| Bq4_CHUSS_What is the most effective way to train health care workers in the prevention of hospital-acquired infections | 20 | 20 |
| combined theoretical and practical training | 8 | 8 |
| convey importance of IPC | 1 | 1 |
| debates on behaviour and risks | 1 | 1 |
| practical training | 4 | 4 |
| routinely | 2 | 2 |
| theoretical training | 1 | 1 |
| through observation of practices and amendment | 1 | 1 |
| use facility infection data | 0 | 0 |
| use staff meetings | 1 | 1 |
| Bq4_DRC_What is the most effective way to train health care workers in the prevention of hospital-acquired infections (2) | 13 | 13 |
| combined theoretical and practical training | 1 | 1 |
| departmental follow-up | 1 | 1 |
| importance of masks | 1 | 1 |
| institutional meetings | 1 | 1 |
| organize trainings at any time | 0 | 0 |
| to also consider awareness and responsibility | 2 | 2 |
| using active or participatory methods | 2 | 2 |
| FUq4_CHUSS_What is the most effective way to train health care workers in the prevention of hospital-acquired infections | 24 | 24 |
| combined theoretical and practical training | 13 | 13 |
| convey exposure risks | 1 | 1 |
| ensure everyone gets training | 3 | 3 |
| involvement in entire IPC program process | 2 | 2 |
| practical training | 5 | 5 |
| staff meetings or bed-side | 1 | 1 |
| through motivation and incentives | 1 | 1 |
| through observation of practices and amendment | 1 | 1 |
| FUq4_DRC_What is the most effective way to train health care workers in the prevention of hospital-acquired infections (2) | 10 | 10 |
| annual training | 1 | 1 |
| cards about HH and mask awareness | 4 | 4 |
| information with empowerment and evaluation | 1 | 1 |
| intercative and participative training | 1 | 1 |
| process integration from the beginning of the action | 1 | 1 |
| standard and complementary precautions | 1 | 1 |

Codes\\Open-Ended Questions\\Q4 Sub-Themes\\Q4 Themes

| Name | Files | References |
| --- | --- | --- |
| Bq4_CHUSS_What is the most effective way to train health care workers in the prevention of hospital-acquired infections | 20 | 20 |
| practical or theoretical training | 0 | 0 |
| combined theoretical and practical training | 8 | 8 |
| practical training | 4 | 4 |
| theoretical training | 1 | 1 |
| Practical or theoretical training, ideally with context-specific content (with majority proposing both) | 0 | 0 |
| through observation of practices and amendment | 1 | 1 |
| use facility infection data | 1 | 1 |
| raised awareness through information dissemination (i.e. importance of measures, risks, responsbilities) | 0 | 0 |
| convey importance of IPC | 1 | 1 |
| debates on behaviour and risks | 1 | 1 |
| routinely or in staff meetings | 0 | 0 |
| routinely | 2 | 2 |
| use staff meetings | 1 | 1 |
| Bq4_DRC_What is the most effective way to train health care workers in the prevention of hospital-acquired infections (2) | 13 | 13 |
| instituionally or departmentally at any time | 0 | 0 |
| departmental follow-up | 1 | 1 |
| institutional meetings | 1 | 1 |
| organize trainings at any time | 1 | 1 |
| raised awareness through information dissemination (i.e. importance of measures, risks, responsbilities) | 0 | 0 |
| importance of masks | 1 | 1 |
| to also consider awareness and responsibility | 2 | 2 |
| through participatory methods or combined theoretical and practical training | 0 | 0 |
| combined theoretical and practical training | 1 | 1 |
| using active or participatory methods | 2 | 2 |
| FUq4_CHUSS_What is the most effective way to train health care workers in the prevention of hospital-acquired infections | 24 | 24 |
| Ensure everyone receives training, ideally on an annual basis with standard and complementary precautions | 3 | 3 |
| involvement in entire IPC program process | 2 | 2 |
| practical or theoretical training | 0 | 0 |
| combined theoretical and practical training | 13 | 13 |
| practical training | 5 | 5 |
| raised awareness through information dissemination (i.e. importance of measures, risks, responsbilities) | 0 | 0 |
| convey exposure risks | 1 | 1 |
| through motivation and incentives | 1 | 1 |
| staff meetings or bed-side | 1 | 1 |
| through observation of practices and amendment | 1 | 1 |
| FUq4_DRC_What is the most effective way to train health care workers in the prevention of hospital-acquired infections (2) | 10 | 10 |
| Ensure everyone receives training, ideally on an annual basis with standard and complementary precautions | 0 | 0 |
| annual training | 1 | 1 |
| standard and complementary precautions | 1 | 1 |
| participative training or process integration from the beginning of the action | 0 | 0 |
| interactive and participative training | 1 | 1 |
| process integration from the beginning of the action | 1 | 1 |
| raised awareness through information dissemination (i.e. importance of measures, risks, responsbilities) | 0 | 0 |
| cards about HH and mask awareness | 4 | 4 |
| information with empowerment and evaluation | 1 | 1 |

Codes\\Open-Ended Questions\\Q5 Sub-Themes

| Name | Files | References |
| --- | --- | --- |
| Bq5_CHUSS_How can you use hospital-acquired infection surveillance data (2) | 19 | 19 |
| assess hygiene level | 1 | 1 |
| behavioral change | 4 | 4 |
| compare between services | 1 | 1 |
| evaluate effectiveness of IPC interventions | 3 | 3 |
| feedback | 4 | 4 |
| improve or modify measures and protocols | 2 | 2 |
| improve quality of care and control HAI | 6 | 6 |
| mortality reduction | 1 | 1 |
| raise awareness | 1 | 1 |
| scientific purposes | 1 | 1 |
| Bq5_DRC_How can you use hospital-acquired infection surveillance data | 13 | 13 |
| as a indicator for quality of care or hygiene | 2 | 2 |
| can only be used if have collection tools | 4 | 4 |
| for monitoring and control | 1 | 1 |
| to give feedback inform decision making | 2 | 2 |
| to respect HH measures | 1 | 1 |
| training | 2 | 2 |
| FUq5_CHUSS_How can you use hospital-acquired infection surveillance data | 24 | 24 |
| as feedback for behavioral change | 2 | 2 |
| for training and decision making | 2 | 2 |
| to advocate for support | 1 | 1 |
| to correct or improve IPC implementation | 7 | 7 |
| to evaluate IPC programme effectiveness | 4 | 4 |
| to formulate an action plan | 1 | 1 |
| to improve quality of care | 1 | 1 |
| to motivate IPC actors | 1 | 1 |
| to reduce cost of hospitalization | 1 | 1 |
| to reduce infection, morbidity and mortality | 3 | 3 |
| to set intervention alerts | 1 | 1 |
| FUq5_DRC_How can you use hospital-acquired infection surveillance data (2) | 10 | 10 |
| as a quality assurance indicator | 2 | 2 |
| guide IPC implementation | 2 | 2 |
| to detect outbreaks | 1 | 1 |

Codes\\Open-Ended Questions\\Q5 Sub-Themes\\Q5 themes

| Name | Files | References |
| --- | --- | --- |
| Bq5_CHUSS_How can you use hospital-acquired infection surveillance data (2) | 19 | 19 |
| by giving feedback and raising awareness for behavoiral change | 0 | 0 |
| behavioral change | 4 | 4 |
| feedback | 4 | 4 |
| raise awareness | 1 | 1 |
| to assess and evaluate effectiveness of IPC interventions | 0 | 0 |
| assess hygiene level | 1 | 1 |
| compare between services | 1 | 1 |
| evaluate effectiveness of IPC interventions | 3 | 3 |
| scientific purposes | 1 | 1 |
| to improve IPC measures and quality of care | 0 | 0 |
| improve or modify measures and protocols | 2 | 2 |
| improve quality of care and control HAI | 6 | 6 |
| mortality reduction | 1 | 1 |
| Bq5_DRC_How can you use hospital-acquired infection surveillance data | 13 | 13 |
| as an indicator to monitor hygiene or quality of care | 0 | 0 |
| as a indicator for quality of care or hygiene | 2 | 2 |
| for monitoring and control | 1 | 1 |
| to respect HH measures | 1 | 1 |
| as feedback that can inform decision making and trainings | 0 | 0 |
| to give feedback inform decision making | 2 | 2 |
| training | 2 | 2 |
| can only be used if have collection tools | 4 | 4 |
| FUq5_CHUSS_How can you use hospital-acquired infection surveillance data | 24 | 24 |
| as feedback for behavoiral change, training and decision making | 0 | 0 |
| as feedback for behavioral change | 2 | 2 |
| for training and decision making | 2 | 2 |
| to motivate IPC actors | 1 | 1 |
| to set intervention alerts | 1 | 1 |
| to evaluate and improve IPC programmes and quality of care | 0 | 0 |
| to improve IPC programme implementation and evaluate effectiveness | 0 | 0 |
| to correct or improve IPC implementation | 7 | 7 |
| to evaluate IPC programme effectiveness | 4 | 4 |
| to formulate an action plan | 1 | 1 |
| to improve quality of care | 1 | 1 |
| to reduce infection, morbidity and mortality | 3 | 3 |
| to reduce cost and advocate for support | 0 | 0 |
| to advocate for support | 1 | 1 |
| to reduce cost of hospitalization | 1 | 1 |
| FUq5_DRC_How can you use hospital-acquired infection surveillance data (2) | 10 | 10 |
| as a quality assurance indicator | 2 | 2 |
| guide IPC implementation | 2 | 2 |
| to detect outbreaks | 1 | 1 |

Codes\\Plenary Codes by CC

| Name | Files | References |
| --- | --- | --- |
| CC 1 IPC programme | 2 | 3 |
| Employment of 100% Focal Point is Problematic | 2 | 2 |
| Insufficient commitment is an obstacle to IPC programmes | 1 | 3 |
| Limited Resources are an obstacle to IPC programmes | 1 | 2 |
| Low awareness or misperception is an obstacle to IPC Programmes | 2 | 4 |
| Organizational issues and non-functional committee are an obstacle to IPC programmes | 1 | 3 |
| Renewal of Patient Escorts is an obstacle to IPC Programmes | 1 | 1 |
| Responsible Health Unit for IPC is Unclear | 1 | 1 |
| CC 2 IPC guidelines | 1 | 1 |
| insufficient implementation of protocols and procedures is a chllenge to CC2 | 1 | 1 |
| Insufficient involment and communication between different actors is a challenge to CC2 | 1 | 2 |
| Insufficient protocols and procedures are a challenge to CC2 | 1 | 1 |
| CC 3 IPC education and training | 1 | 1 |
| including trainee care givers in IPC training is problematic to CC3 | 1 | 1 |
| CC 4 HAI surveillance | 1 | 1 |
| insufficient data collection and reporting is a barrier to CC4 | 1 | 2 |
| Limited resources are a barrier to CC4 | 1 | 4 |
| CC 5 Multimodal strategies | 1 | 1 |
| attitudes and knowledge are a barrier to CC5 | 1 | 3 |
| insufficient promotion of IPC is a barrier to CC5 | 1 | 1 |
| Limited resources are a barrier to CC5 | 1 | 2 |
| CC 6 Monitoring, audits of IPC practices and feedback | 1 | 1 |
| lack of training, audit programmes and resulting feedback | 1 | 3 |
| limited resources are a barrier to CC6 | 1 | 1 |
| low involvement of decision-makers | 1 | 1 |
| CC 7 Workload, staffing and bed occupancy | 1 | 1 |
| limited resources are a barrier to CC7 | 1 | 2 |
| Organizational Issues are a barrier to CC7 | 1 | 4 |
| overcrowding is a barrier to CC7 | 1 | 1 |
| Unforeseen Events are a barrier to CC7 | 1 | 1 |
| CC 8 Built environment, materials and equipment for IPC at the facility level | 3 | 10 |
| limited resources are a barrer to CC8 | 2 | 17 |
| non-compliance is a barrier to CC8 | 1 | 1 |
| water is an essential component of CC8 | 2 | 5 |
| General IPC | 3 | 8 |
| attitudes and knowledge are a barrier to IPC | 1 | 1 |
| Infections are spread via direct and indirect contact | 1 | 4 |
| IPC is important for many reasons that benefit both HCW and patients | 1 | 4 |
| lack of COVID-19 triage | 1 | 1 |

Codes\\Plenary Codes by CC\\Plenary Themes by CC

| Name | Files | References |
| --- | --- | --- |
| CC 1 IPC programme | 2 | 3 |
| attitudes are a barrier (including awareness, misperceptions and commitment) | 0 | 0 |
| Insufficient commitment is an obstacle to IPC programmes | 1 | 3 |
| Low awareness or misperception is an obstacle to IPC Programmes | 2 | 4 |
| Limited resources are a barrier (including human resources) | 0 | 0 |
| Employment of 100% Focal Point is Problematic | 2 | 2 |
| Limited Resources are an obstacle to IPC programmes | 1 | 2 |
| Organizational issues and unclear responsibilites are a barrier | 0 | 0 |
| Organizational issues and non-functional committee are an obstacle to IPC programmes | 1 | 3 |
| Responsible Health Unit for IPC is Unclear | 1 | 1 |
| Renewal of Patient Escorts is an obstacle to IPC Programmes | 1 | 1 |
| CC 2 IPC guidelines | 1 | 1 |
| Insufficient involment and communication between actors are barriers | 1 | 2 |
| Insufficient protocols and procedures and implementation thereof are barriers | 0 | 0 |
| insufficient implementation of protocols and procedures is a chllenge to CC2 | 1 | 1 |
| Insufficient protocols and procedures are a challenge to CC2 | 1 | 1 |
| CC 3 IPC education and training | 1 | 1 |
| including trainee care givers in IPC training is problematic to CC3 | 1 | 1 |
| CC 4 HAI surveillance | 1 | 1 |
| insufficient data collection and reporting is a barrier to CC4 | 1 | 2 |
| Limited resources are a barrier to CC4 | 1 | 4 |
| CC 5 Multimodal strategies | 1 | 1 |
| attitudes and knowledge are a barrier to CC5 | 1 | 3 |
| insufficient promotion of IPC is a barrier to CC5 | 1 | 1 |
| Limited resources are a barrier to CC5 | 1 | 2 |
| CC 6 Monitoring, audits of IPC practices and feedback | 1 | 1 |
| lack of training, audit programmes and resulting feedback | 1 | 3 |
| limited resources are a barrier to CC6 | 1 | 1 |
| low involvement of decision-makers | 1 | 1 |
| CC 7 Workload, staffing and bed occupancy | 1 | 1 |
| limited resources are a barrier to CC7 | 1 | 2 |
| Organizational Issues are a barrier to CC7 | 1 | 4 |
| overcrowding is a barrier to CC7 | 1 | 1 |
| Unforeseen Events are a barrier to CC7 | 1 | 1 |
| CC 8 Built environment, materials and equipment for IPC at the facility level | 3 | 10 |
| limited resources are a barrer to CC8 | 2 | 17 |
| non-compliance is a barrier to CC8 | 1 | 1 |
| water is an essential component of CC8 | 2 | 5 |
| General IPC | 3 | 8 |
| attitudes and knowledge are a barrier to IPC | 1 | 1 |
| Infections are spread via direct and indirect contact | 1 | 4 |
| IPC is important for many reasons that benefit both HCW and patients | 1 | 4 |
| lack of COVID-19 triage | 1 | 1 |

Codes\\Plenary Data_CHUSS

| Name | Files | References |
| --- | --- | --- |
| CC5 Multimodal Strategies | 0 | 0 |
| CC5 barrier is insuff promotion of IPC | 1 | 4 |
| CC5 barrier is lack of resources | 1 | 2 |
| CC5 barrier is low importance of IPC due to lack of training | 1 | 1 |
| CC5 barrier is negative individual attitudes to system change | 1 | 1 |
| CC5 low staff mobilization | 1 | 1 |
| HAI surveillance barrier is cost of complementary examinations | 1 | 1 |
| HAI surveillance barrier is inadequate reporting | 1 | 1 |
| HAI surveillance barrier is insuff data collection | 1 | 1 |
| HAI surveillance barrier is lack of equipement | 1 | 1 |
| HAI surveillance barrier is lack of financial resources | 1 | 1 |
| HAI surveillance barrier is lack of human resources | 1 | 1 |
| HH is important for providing safe care | 1 | 1 |
| HH is important to eliminate self-contamination and transmission of germs | 1 | 1 |
| HH via ABHR | 1 | 2 |
| HH via soap and water | 1 | 2 |
| HH via surgical washing | 1 | 1 |
| Infections are spread via direct contact | 1 | 4 |
| infections are spread via indirect contact | 1 | 3 |
| Infections are spread via noncompliance | 1 | 2 |
| infections are spread via patient attendants | 1 | 1 |
| IPC Guideline challenges are insuf implementation | 1 | 1 |
| IPC Guideline challenges are insuff production of evidence | 1 | 1 |
| IPC Guideline challenges are insufficient protocols and procedures | 1 | 1 |
| IPC guideline challenges are low involvement of actors in implementation | 1 | 1 |
| IPC guideline challengese are insuff communication | 1 | 1 |
| IPC is a set of procedures, rules, measures, strategies and practices to prevent the occurrence, transmission and spread of HAI | 1 | 4 |
| IPC is important because it can break the chain of transmission of infections at the individual and collective level | 1 | 4 |
| IPC is important because it can improve quality of care | 1 | 1 |
| IPC is important because it can Improve working and living conditions | 1 | 1 |
| IPC is important because it can lead to behavioral change | 1 | 2 |
| IPC is important because it can protect HCW and patient health | 1 | 2 |
| IPC is important because it can reduce adverse effects (HAIs) | 1 | 3 |
| IPC is important because it can reduce length of hospital stay and mortality and the spread of multi-drug resistant bacteria | 1 | 1 |
| IPC is important because it can reduce workload | 1 | 1 |
| IPC is important beuase it can result in proper waste management | 1 | 1 |
| IPC Prog Obst is limited finanicial resources | 1 | 2 |
| IPC Prog Obstacle is lack of management committment | 1 | 1 |
| IPC Prog Obstacle is lack of staff committment | 1 | 2 |
| IPC Prog Obstacle is low awareness | 1 | 2 |
| IPC Prog Obstacle is Misperception of the importance of PCI | 1 | 1 |
| IPC prog obstacle is non-functional IPC committee | 1 | 1 |
| IPC Prog Obstacle is patient escorts | 1 | 1 |
| IPC Prog Obstacles are lack of material and facility resources | 1 | 1 |
| IPC Progr Obstacles are Organizational problems | 1 | 2 |
| IPC program obstacles are lack of trained focal points | 1 | 1 |
| water is life and needed for many uses | 1 | 1 |
| water need for hosp patient (24hrs) is 143L | 1 | 1 |
| water need for outpatient is 9L | 1 | 2 |
| water supply sources are wells, running water, and boreholes | 1 | 1 |

Codes\\Plenary Data_DRC

| Name | Files | References |
| --- | --- | --- |
| Confusion in differences between IPC committee and Hygiene committee | 1 | 1 |
| Hand washing with ash because of soap and water shortage | 1 | 1 |
| Improper instrument sterlization with chlorine | 1 | 1 |
| IPC new concept only for during epidemics | 1 | 1 |
| It is problematic to have a 100% dedicated focal point without clinical duties | 1 | 1 |
| lack of COVID-19 triage | 1 | 1 |
| multiple patient use of baxter infusion sets | 1 | 1 |
| New quarterly trainees (HCWs) not included in training sessions because they are not considered to be employees | 1 | 1 |
| Only Busy rooms with high patient traffic are being disinfected | 1 | 1 |
| The abcense of a national IPC program is reflected a the provincial and health zone levels | 1 | 1 |
| the Hygiene committee should receive IPC perogatives | 1 | 1 |
| the MoH guidelines provide for a hygiene commitee but not an IPC committee | 1 | 1 |
| Usage of cloth masks of to shortage | 1 | 1 |
| Use of local disinfectant with no compositionindication | 1 | 1 |
