# Supplementary material for: Implementation of the WHO core components of an infection prevention and control programme in two sub-saharan African acute health-care facilities: a mixed methods study
Source: Antimicrob Resist Infect Control. 2024 Jan 15;13:4. doi: 10.1186/s13756-023-01358-1 (PMC10789048; doi:10.1186/s13756-023-01358-1)
Supplement: Supplementary file 3 — Supplementary Material 3 [file 13756_2023_1358_MOESM3_ESM.docx]

Additional file 1: Mental maps of West Africa, socio-spatial patterns

**
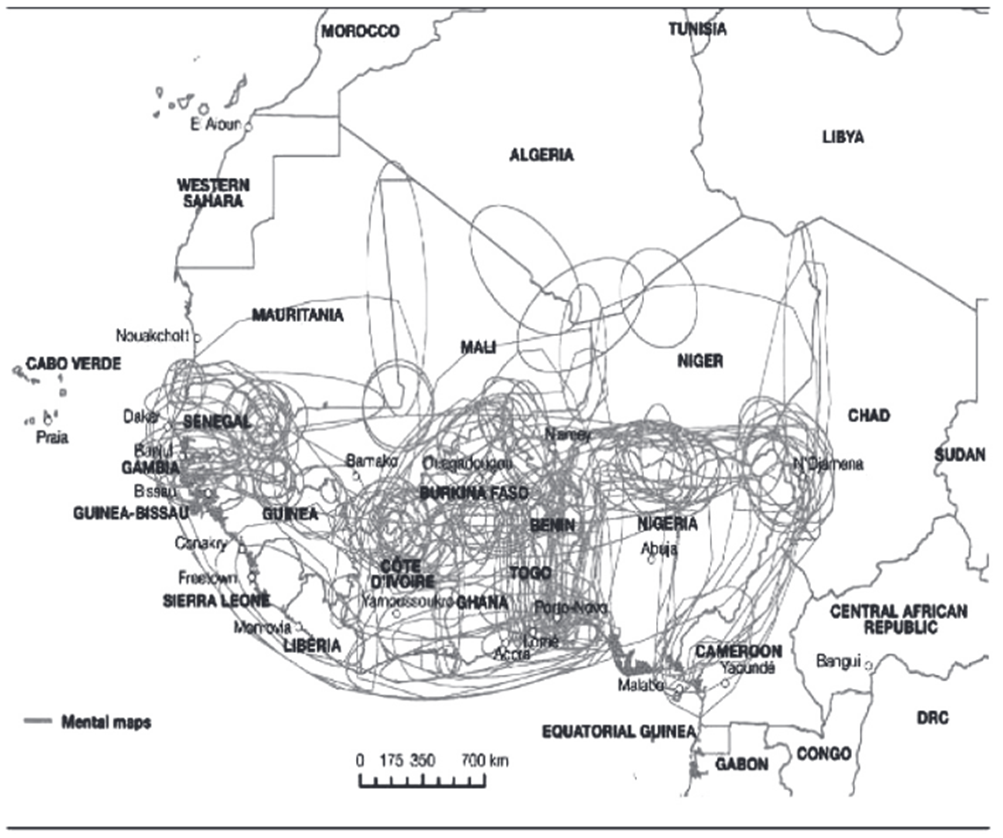
**

Reference: Faleye, O.A., *Sociospatial Networks and Transborder Epidemic Surveillance in West Africa: A Review of the Ebola Outbreak, 2014-2015.* The Nigerian Health Journal. **17.3**: p. 61-69.
